# Soluble Urokinase Plasminogen Activator Receptor Levels Correlation with Other Inflammatory Factors in Prognosis of Disability and Death in Patients with Ischemic Stroke

**DOI:** 10.3390/brainsci12010039

**Published:** 2021-12-28

**Authors:** Dorota Różański, Stanisław Szlufik, Ryszard Tomasiuk, Łukasz Milanowski, Monika Figura, Kamila Saramak, Piotr Myrcha, Dariusz Koziorowski

**Affiliations:** 1 Department of Neurology, Faculty of Health Sciences, Medical University of Warsaw, 02-091 Warsaw, Poland; lukasz.milanowski@wum.edu.pl (Ł.M.); monika.figura@wum.edu.pl (M.F.); kamilasaramak@gmail.com (K.S.); dkoziorowski@wum.edu.pl (D.K.); 2 Faculty of Medical Sciences and Health Sciences, Kazimierz Pulaski University of Technology and Humanities, 26-600 Radom, Poland; sszlufik@wum.edu.pl (S.S.); r.tomasiuk@wp.pl (R.T.); 3 Department of General and Vascular Surgery, Faculty of Medicine, Medical University of Warsaw, 02-091 Warsaw, Poland; piotr.myrcha@wum.edu.pl; 4 Department of General, Vascular and Oncological Surgery, Masovian Brodnowski Hospital, 03-242 Warsaw, Poland

**Keywords:** ischemic stroke, suPAR, cardiovascular disease, inflammation, atherosclerosis, carotid artery disease

## Abstract

Soluble urokinase plasminogen activator receptor (suPAR) is an inflammatory biomarker elevated in cardiovascular diseases. The aim of this 3-year follow-up prospective study was to evaluate suPAR levels in patients with a first ischemic stroke in correlation with CRP, PCT, NT-proCNP and endothelin 1-21 and to investigate the impact of suPAR on the outcome. Fifty-one patients (mean age 73.7+ = 11.9 years, 26 female and 25 male) were included. Samples were collected on the first (suPAR 1), third (suPAR 3) and seventh days after stroke onset (suPAR 7). Plasma samples were analyzed using ELISA. A phone interview was conducted to collect follow-up information after 24 and 36 months (modified Rankin Scale, mRS). A positive correlation between suPAR levels and other inflammatory biomarkers (except endothelin 3) was observed. A positive correlation between suPAR 3 and mRS score at 24 months was observed *(p* = 0.042). The logistic regression model revealed no significant effect of suPAR on death occurrence in the first 24 months: suPAR 1 (*p* = 0.8794), suPAR 3 (*p* = 0.2757), and suPAR 7 (*p* = 0.3652). The suPAR level is a potential inflammatory marker in ischemic stroke, and there is a correlation with other markers. There is no major impact on mortality. However, the suPAR level is associated with a degree of disability or dependence in daily activities 2 years after a stroke.

## 1. Introduction

Over the last few decades, increased evidence has suggested that chronic inflammation plays a major role in the development of atherosclerosis [1,2]. Therefore, several population-based studies have investigated the relationship between a number of inflammatory biomarkers and cardiovascular disease (CVD), including ischemic stroke [3,4,5].

Soluble urokinase plasminogen activator receptor (suPAR) is an emerging biomarker which is thought to reflect the activation of the immune system [6]. It is a soluble bioactive form of urokinase plasminogen activator receptor (uPAR, CD 87), which is a membrane protein located on various types of cells, including monocytes, macrophages, activated T-lymphocytes, endothelial and smooth muscle cells [7,8,9,10,11,12,13], all of which are involved in atherosclerosis [14]. Not only is the uPA–uPAR system known to be involved in fibrinolysis through the plasminogen activating pathway, but it also regulates angiogenesis, cell proliferation, migration, adhesion and recruitment of inflammatory cells [15,16,17]. Soluble uPAR is cleaved from the cell membranes and released into the circulation due to either acute or chronic inflammation [6,18].

High plasma levels of suPAR were detected in patients with infectious diseases [19,20] as well as several types of cancer [21,22,23,24] and are associated with poor prognosis. Lately, suPAR has gained interest as a possible risk marker for CVD, type 2 diabetes mellitus (DM2), cancer and mortality in the general population [25,26,27]. Elevated levels of suPAR in plasma have also been linked to subclinical organ damage and cardiovascular events, helping to predict all-cause mortality in transient ischemic attack (TIA) and ischemic stroke [27,28]. Few studies focused on the evaluation of suPAR levels in plasma in association with the histopathological assessment of atherosclerotic plaque morphology [26]. A study by Onatsu et al. showed that suPAR concentration was higher in patients after a stroke/TIA due to large-artery atherosclerosis compared to small-vessel disease (SVD), and that elevated plasma suPAR concentrations predicted all-cause mortality during the 5-year follow-up [29].

A study by Kiiski et al. reported that plasma suPAR is not associated with neurological outcome after subarachnoid hemorrhage, but there is a lack of publications that observe the correlation between suPAR level and outcome after ischemic stroke [30].

The aim of this 3-year follow up prospective study was to evaluate suPAR levels in patients with a first-ever ischemic stroke in correlation with established inflammatory markers—C-reactive protein (CRP), procalcitonin (PCT), n-terminal pro c-type natriuretic peptide (NT-proCNP) and markers of endothelial damage (endothelin 1-21, NT-proCNP)—and to investigate if elevated levels of suPAR are associated with the degree of disability and death in patients with ischemic stroke, assessed on a modified Rankin Scale (mRS).

## 2. Materials and Methods

### 2.1. Patients

Fifty-one patients (mean age of 73.7 ± 11.9 years, 26 female and 25 male) with a first-ever ischemic stroke confirmed by computer tomography (CT) scan (CT GE light speed 8, layer thickness 2.5 mm) and/or magnetic resonance imaging (MRI) of the brain (MRI GE Sigma excite 1.5T) were included in the study.

Patients had the following risk factors for stroke: hypertension (40 = 78.43%), diabetes (19 = 37.25%), hyperlipidemia (25 = 49.01%), coronary artery disease (11 = 21.56%), history of myocardial infarction (7 = 13.72%), heart failure (3 = 5.88%), atrial fibrillation (16 = 31.37%), and smoking (7 = 13.72%) (Table 1).

Considering the risk factors for stroke and the results of neuroimaging, the patients were classified according to the TOAST classification.

The patients were treated with the standard therapy of ischemic stroke, involving thrombolytic therapy, antiplatelet therapy, anticoagulant therapy, antihypertensive treatment, treatment of heart failure, and lipid-lowering and hypoglycemic treatment (Table 1).

Patients were recruited in the Department of Neurology, Masovian Brodnowski Hospital, Warsaw, between years 2013 and 2014. Exclusion criteria included detection of hemorrhagic stroke on imaging, generalized neoplastic disease, sepsis, previous history of stroke and a second stroke during hospitalization. During hospitalization, all patients received routine treatment for the secondary prevention of stroke. A total of 9 out of 51 patients developed pneumonia, and 7 out of 51 a urinary tract infection during hospitalization. In total, 1 patient developed a gastrointestinal tract infection. 

Doppler ultrasonography (DUS) of the carotid and vertebral arteries was performed to assess the presence of atherosclerotic lesion. DUS was performed according to the protocol of the Polish Ultrasound Society using the Philips HD 15 ultrasound machine with a 10–12 Hz linear probe. Atherosclerotic lesions in the carotid arteries were visualized in 36 (70.6%) patients. Significant stenosis of internal carotid artery (greater than 50%) was found in 15 patients (29.4%), including occlusion in 5 patients (9.8%). Bilateral stenoses were found in 6 patients (11.7%). One patient underwent carotid endarterectomy without complication.

Twenty-four-hour Holter electrocardiograph (ECG) monitoring and/or ECG examination were performed to detect atrial fibrillation and other cardiac arrhythmias. A detailed medical history of each patient was collected. In addition to specific inflammation biomarkers, lipid profile, renal function parameters, fasting glucose measurement and hematological parameters were assessed.

The type of ischemic stroke was assessed according to the TOAST classification [31]. The etiology according to TOAST classification included cardioembolism in 13 patients (26%), large-artery atherosclerosis in 16 patients (32%), small-vessel disease in 8 patients (16%). Thirteen patients (26%) had cryptogenic stroke with unidentified cause.

After 24 months and 36 months from discharge, the patients or their relatives were contacted by phone (K.S., D.R.) and evaluated with the mRS. Information was gathered about follow-up end points, recurrent strokes, level of disability and death.

All participants provided informed signed consent for participation in the study. The study was approved by the Ethics Review Board of Medical University of Warsaw (KB/211/2013).

### 2.2. Biochemical Measurements 

Blood samples were collected on the first (suPAR1), third (suPAR3) and seventh (suPAR7) days after stroke onset. After collection, the samples were centrifuged and immediately frozen to −20 degrees Celsius. Apart suPAR concentration the plasma samples were also analyzed on the first, third and sevenths days after stroke onset for CRP, PCT, endothelin and NT-proCNP plasma levels using enzyme-linked immunosorbent assay (ELISA). 

Serum cytokines were measured using the fluorokine^®^ MAP cytokine multiplex kit and the LuminexTM 100 Platform. Microglobules marked fluorescently with specific antibodies were positioned along with standards and serum samples examined in pits. The serum cytokines were incubated with antibodies. Afterwards, biotin conjugated antibodies were added to combine with antibody-linked cytokines. After subsequent rinsing, streptavidin-PE conjugate was infused to connect with biotin and to emit fluorescent signal. After one more rinsing, to remove excess streptavidin, micromolecules were placed in a buffer. The 2-laser analysis of the buffer was performed on the LuminexTM 100 Platform software in accordance with to the procedures supplied by the manufacturer. 

### 2.3. Statistics

All statistical analyses were performed with Statistica 13. Normality of distribution was assessed using the Shapiro–Wilk test. Continuous variables are presented as average and standard deviations. Categorical variables are presented as percentages. Correlation with other inflammatory biomarkers and with mRS score was estimated using a Pearson correlation test. A univariate, independent logistic regression model was conducted to evaluate the effect of suPAR on death occurrence in the first 24 months after ischemic stroke. What is more, the least absolute shrinkage and selection operator (LASSO) regression model was conducted to evaluate the effect of suPAR concentration on disability scales and various clinical factors. The estimation was performed based on the covariance actualization. Predictors with stable values were removed from the model. Kaplan–Meier analysis was performed to present survival rate during 36 months observation time. A value of *p* ≤ 0.05 was considered significant for all tests.

## 3. Results

Biochemical data of the patients are presented in Table 2. The mean level of suPAR 1/2/3 was 3.43 ± 2,2/3.58 ± 3,0/4.22 ± 3,9 mg/mL. The serum levels of suPAR 1/3/7 were strongly correlated with the serum levels of PCT 1/3/7 (R1 = 0.96/R2 = 0.96/R3 = 0.97, *p* < 0.05) and the serum level of NT-proCNP 1/3/7 (R1 = 0.78/R2 = 0.77/R3 = 0.92, *p* < 0.05). 

A positive correlation between suPAR 3 and mRS at 24 months was observed (*p* = 0.042) (Table 3). A logistic regression model revealed no significant effect of suPAR on mortality in the first 24 months (suPAR1 (*p* = 0.879), suPAR3 (*p* = 0.275), suPAR7 (*p* = 0.365). What is more, LASSO penalized regression model revealed no effect of suPAR on different clinical parameters and disability scales (Table 4). 

A non-significant effect was observed for suPAR on death difference between suPAR 1 and suPAR 3 (*p* = 0.108) and between suPAR 1 and suPAR 7 (*p* = 0.802).

No correlation was observed between suPAR 1, 3, and 7 and LAO and other types of strokes.

**Figure 1:**
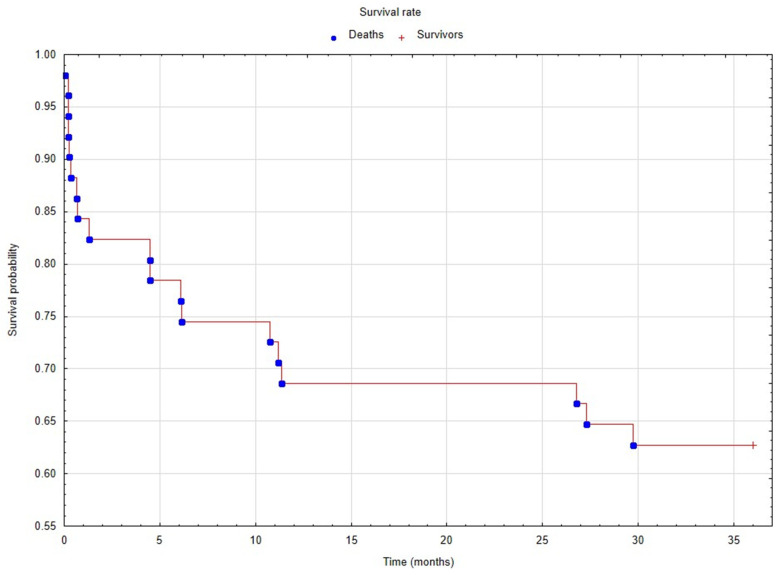
Survival rate in the analyzed population during 36 months of observation. The Kaplan–Meier estimates were performed to present the survival probability in patients with stroke. The estimated interquartile range for the survival was between 6.13 and 36 months, and survival rate was 62.7% (blue circle—ischemic stroke patients dead during 36-month observation time; red cross—ischemic stroke patients survived during 36-month observation time). The SUPAR 1, 3 and 7 concentration for LAO was 3.6 ± 3.0, 3.8 ± 4.9 and 4.7 ± 6.3 respectively, for lacunar 3.2 ± 1.1, 2.6 ± 1.3 and 4.1 ± 2.0 respectively, for cardioembolic 3.2 ± 2.3, 3.1 ± 1.9 and 3.4 ± 2.6 respectively and for ESUS 3.5 ± 1.5, 3.6 ± 1.9 and 2.9 ± 2.0 respectively.

In the 3-year observation study, there were 13 recurrent strokes (12 during the 24-month and an additional one during the 36-month follow-up). High mortality was observed. A total of 19 patients died (37.3%): 16 during the first 24 months (31.4%) and additional 3 up to 36 months (6%). The Kaplan–Meier curve is presented in Figure 1; the interquartile range for the survival was between 6.13 and 36 months.

## 4. Discussion

Increased concentration of suPAR is associated in the literature with the development, severity, and outcome of diseases, such as sepsis, tuberculosis, cancer, diabetes mellitus or renal disease [19,25]. suPAR is proposed as a biomarker of acute and chronic organ damage. It was, however, especially investigated as a biomarker of low-grade inflammation, which is observed in cardiovascular diseases, such as stroke or coronary artery disease (CAD) [32]. It was documented that increased levels of suPAR coincide with endothelium dysfunction, vascular stiffness and worsened cardiac microcirculation or atherosclerosis. Numerous studies investigated its utility as a predictor of outcome or biomarker of severity of stroke, but the results are equivocal. In our study, we assessed if suPAR levels correlate with the outcome of stroke measured in the mRS of disability. We also wanted to investigate if suPAR concentrations are dependent on the stroke subtype in the TOAST classification. Our findings confirm that higher concentrations of suPAR coincide with higher level of disability measured in the mRS.

A few studies investigated potential correlations between suPAR concentration and stroke outcomes. Currently, numerous methods are applied, including imaging, sociodemographic factors and biochemical markers to predict stroke outcome [33,34]. suPAR levels are reported by some authors as being elevated in atherosclerotic etiology of the stroke. Persson et al. reported the relationship between serum suPAR and the occurrence of carotid plaques and the incidence of stroke and CAD. Patients with high suPAR and carotid plaque had an hazard ratio (HR) of 2.2 (95% CI 1.5–3.2) for ischemic stroke and HR of 1.7 (95% CI 1.2–2.4) for CAD [27]. The results of this study are of special importance because of the large study group (5166 people). A study by Edsfeldt et al. reported increased levels of suPAR in plasma and atherosclerotic plaque homogenate of patients with symptomatic LAO due to atherosclerosis, compared to asymptomatic patients [26]. In the present study, it was observed that suPAR concentration fails to differentiate between stroke due to LAO (in all cases caused by symptomatic atherosclerosis) or to other causes. This is partially in line with the findings of Onatsu et al. [29]. The authors reported that although elevated suPAR allows prediction of all-cause mortality due to stroke, it does not differentiate between all stroke types. They reported, however, that patients with stroke due to LAO had a significantly higher suPAR level than those with SVD. In the present study, no correlation was observed between concentrations of suPAR 1, 3, and 7 and types of strokes classified according to TOAST classification. This may be explained by the fact that elevated suPAR levels are not specific for atherosclerosis but may also reflect the activation of numerous inflammatory systems. This may be due to the infectious complications of stroke, which are frequent among patients with stroke.

suPAR should also be taken into consideration as a possible prognostic factor of functional disability evaluated with the mRS and other function outcomes. In the present study, a significant positive correlation between suPAR and the mRS was observed, between suPAR 3 measured on the third day after stroke onset and the mRS 24 months from stroke onset. To the best of our knowledge, while many papers investigated prognostic factors of stroke outcome [35], this is the first study to investigate correlations between suPAR levels in ischemic stroke and the functional outcome of patients with a 3-year long follow-up. suPAR was proposed as a predictor of 5-year all-cause mortality in a study by Onatsu et al. [29] In the present study, such a correlation was not observed. This may be due to a shorter follow-up (3 vs. 5 years) and a smaller patient group (51 vs. 117). Importantly, our study group included much older patients (73.7 vs. 61 years), which may affect mortality [29]. Additionally, it did not include a group of patients with TIA. This may impact the clinical outcome, especially disability. The mean serum level of suPAR in ischemic stroke patients is correlated with the serum level of PCT (inflammatory marker) and NT-proCNP (inflammatory and endothelial damage marker); therefore, it should be taken into consideration as a possible prognostic factor of inflammation and endothelial damage in this group of patients. The main limitation of the study is the size of the group. 

## 5. Conclusions

The authors conclude that suPAR concentration measured after 3 days from stroke onset can be taken into consideration as a possible prognostic factor of functional disability of patients suffering from stroke due to all causes. suPAR fails to differentiate patients with stroke due to different etiologies. 

## Figures and Tables

**Table 1:** Demographic, epidemiological and pharmacological treatment data.

Number of Patients	51
Males	25
Age: Mean (SD)	73.7(11.9)
**Comorbidities**	**N of patients (%)**
Hypertension	40(78.4)
Diabetes	19(37.2)
Hyperlipidemia	25(49.0)
Coronary artery disease	11(21.6)
Myocardial infarction	7(13.7)
Heart failure	3(5.9)
Atrial fibrillation	16 (31.3)
Smoking	7 (13.7%)
**Pharmacological treatment**	
Thrombolytic therapy	7 (13.7)
Antiplatelet therapy	
ASA 150 mg	28 (54.9)
ASA 75 mg	13 (25.5)
ASA + Clopidogrel	1 (2.0)
Clopidogrel	1 (2.0)
Anticoagulant therapy	
Enoxaparin	15 (29.4)
Dabigatran	4 (7.8)
Antihypertensive/heart failure treatment	
Perindopril	4 (7.8)
Ramipril	7 (13.7)
Enalapril	20 (39.2)
Quinapril	1 (2.0)
Valsartan HCT	1 (2.0)
Amiloride HCT	1 (2.0)
Cilazapril HCT	1 (2.0)
Metoprolol	8 (15.7)
Bisoprolol	15 (29.4)
Carvedilol	1 (2.0)
Amlodipine	13 (25.5)
Nitrendipine	1 (2.0)
Lacidipine	1 (2.0)
Torasemide	2 (3.9)
Indapamide	9 (17.4)
Spironolactone	4 (7.8)
Lipid-lowering treatment	
Simvastatin	28 (54.9)
Atorvastatin	4 (7.8)
Rosuvastatin	1 (2.0)
Fenofibrate	2 (3.9)
Hypoglycemic treatment	
Metformin	8 (15.7)
Glimepiride	4 (7.8)
Insulin	11 (21.6)

ASA—acetylsalicylic acid.

**Table 2:** Biochemical parameter data.

Biochemical Parameter				*p*-Value
	Day after Stroke			
	1st Mean (SD)	3rd Mean (SD)	7th Mean (SD)	
Fibrinogen (mg/dL)	177.44 (169.41)	N/A	N/A	N/A
Urea (mg/dL)	38.51 (17.69)	N/A	N/A	N/A
Creatinine (mg/dL)	0.94 (0.23)	N/A	N/A	N/A
HbA1C (%)	4.85 (2.90)	N/A	N/A	N/A
Cholesterol (mg/dL)	180.59 (52.50)	N/A	N/A	N/A
HDL (mg/dL)	48.35 (13.16)	N/A	N/A	N/A
LDL (mgdL)	107.89 (41.29)	N/A	N/A	N/A
Triglycerides (mg/dL)	128.16 (103.63)	N/A	N/A	N/A
Glucose (mg/dL)	131.94 (52.51)	N/A	N/A	N/A
Inflammatory parameters				
CRP (mg/L)	17.54 (34.30)	36.81 (53.22)	33.74 (43.37)	suPAR 1-3: *p* = 0.0044 suPAR 1-7: *p* = 0.0063
Procalcitonin (ng/mL)	0.31 (1.23)	0.33 (1.08)	0.39 (1.94)	suPAR 1-3: *p* = 0.261 suPAR 1-7: *p* = 0.967
suPAR (ng/mL)	3.42 (2.19)	3.37 (3.04)	3.80 (3.96)	suPAR 1-3: *p*= 0.0865 suPAR 1-7: *p* = 0.2
NT-proCNP (pg/mL)	3.84 (2.23)	4.08 (2.84)	4.37 (4.47)	suPAR 1-3: *p* = 0.0977 suPAR 1-7: *p* = 0.517
Endothelin (pg/mL)	4.03 (14.58)	4.08 (14.53)	N/A	suPAR 1-3: *p* = 0.59

SD—standard deviation; HbA1C—glycated hemoglobin; CRP—c-reactive protein; HDL—high-density lipoprotein; LDL—low-density lipoprotein; suPAR—soluble urokinase plasminogen activator receptor; NT-proCNP—N-terminal pro C-type natriuretic peptide.

**Table 3:** suPAR concentration correlation with disability scales, Pearson coefficient test r (*p*-value).

	mRS Initial N = 51 (*p*-Value)	mRS 24 N = 51 (*p*-Value)	mRS 36 N = 35 (*p*-Value)
suPAR 1st day	0.13 (*p* = 0.37)	0.12 (*p* = 0.4)	−0.30 (*p* = 0.89)
suPAR 3rd day	0.22 (*p* = 0.13)	0.29 (*p* = 0.04)	−0.13 (*p* = 0.48)
suPAR 7th day	0.065 (*p* = 0.65)	0.063 (*p* = 0.66)	−0.02 (*p* = 0.89)

suPAR—soluble urokinase plasminogen activator receptor; mRS 24—mRS score result after 24 months; mRS score 36—result after 36 months; N—number of patients.

**Table 4:** suPAR LASSO regression models with selected clinical data and disability scales.

	suPAR1 − Model Lambda = 0.000642 %Deviation = 0.734219	*p*-Value	suPAR3 − Model Lambda = 0.000569 %Deviation = 0.572278	*p*-Value	suPAR7 − Model Lambda = 0.000640 %Deviation = 0.464577	*p*-Value
	Estimates		Estimates		Estimates	
Age	0.07	0.07	0.08	0.99	0.07	0.86
NIHSS at discharge	0.15	0.15	0.03	0.70	0.02	0.94
mRS 24 months	−0.42	0.39	−0.0002	0.63	0.81	0.90
mRS 36 months	−0.97	0.95	−0.79	0.91	0.07	0.94
Sex	0.09	0.94	−0.33	0.85	0.69	0.94
Hypertension	0.27	0.97	0.33	0.75	0.55	0.95
Diabetes	0.65	0.94	0.62	0.95	−0.11	0.89
AF	−0.03	0.53	−0.37	0.87	−0.26	0.85

NIHSS—National Institute of Health Stroke Scale. AF—atrial fibrillation.

## Data Availability

Data available from the corresponding author on demand.

## List of Abbreviations

suPAR	soluble urokinase plasminogen activator receptor
uPAR	urokinase plasminogen receptor
mRS	modified Rankin Scale
CRP	C-reactive protein
PCT	procalcitonin
NT-proCNP	N-terminal pro C-type natriuretic peptide
ELISA	enzyme-linked immunosorbent assay
CVD	cardiovascular disease
DM2	type 2 diabetes mellitus
CVE	cardiovascular events
TIA	transient ischemic attack
SVD	small vessel disease
CT	computer tomography scan
MRI	magnetic resonance imaging
TOAST	trial of ORG 10172 in acute stroke treatment
ASA	acetylsalicylic acid
ECG	electrocardiograph
LAO	large artery occlusion
ESUS	embolic stroke of undetermined source
SD	standard deviation
HbA1C	glycated hemoglobin
HDL	high-density lipoprotein
LDL	low-density lipoprotein
NIHSS	National Institute of Health Stroke Scale
AF	atrial fibrillation
LASSO	least absolute shrinkage and selection operator
